# Risk of developing severe sepsis after acute kidney injury: a population-based cohort study

**DOI:** 10.1186/cc13054

**Published:** 2013-10-11

**Authors:** Tai-Shuan Lai, Cheng-Yi Wang, Sung-Ching Pan, Tao-Min Huang, Meng-Chun Lin, Chun-Fu Lai, Che-Hsiung Wu, Vin-Cent Wu, Kuo-Liong Chien

**Affiliations:** 1 Department of Internal Medicine, National Taiwan University Hospital, Bei-Hu Branch, 87 Neijiang St, Taipei 108, Taiwan; 2 Institute of Epidemiology and Preventive Medicine, College of Public Health, National Taiwan University, 17 Xu-Zhou Rd, Taipei 100, Taiwan; 3 Department of Internal Medicine, Cardinal Tien Hospital, 362 Zhongzheng Rd, New Taipei City 231, Taiwan; 4 Department of Internal Medicine, National Taiwan University Hospital, 7 Chung-Shan S Rd, Taipei 100, Taiwan; 5 Department of Internal Medicine, National Taiwan University Hospital, Yun-Lin Branch, 579 Sec 2, Yunlin Rd, Douliou City, Yunlin County 640, Taiwan; 6 Department of Internal Medicine, Buddhist Tzu Chi General Hospital, 289 Jianguo Rd, New Taipei City 231, Taiwan

## Abstract

**Introduction:**

Sepsis has been a factor of acute kidney injury (AKI); however, little is known about dialysis-requiring AKI and the risk of severe sepsis after survival to discharge.

**Methods:**

We conducted a population-based cohort study based on the Taiwan National Health Insurance Research Database from 1999 to 2009. We identified patients with AKI requiring dialysis during hospitalization and survived for at least 90 days after discharge, and matched them with those without AKI according to age, sex, and concurrent diabetes. The primary outcome was severe sepsis, defined as sepsis with a diagnosis of acute organ dysfunction. Individuals who recovered enough to survive without acute dialysis were further analyzed.

**Results:**

We identified 2983 individuals (mean age, 62 years; median follow-up, 3.96 years) with dialysis-requiring AKI and 11,932 matched controls. The incidence rate of severe sepsis was 6.84 and 2.32 per 100 person-years among individuals with dialysis-requiring AKI and without AKI in the index hospitalization, respectively. Dialysis-requiring AKI patients had a higher risk of developing de novo severe sepsis than the non-AKI group. In subgroup analysis, even individuals with recovery from dialysis-requiring AKI were at high risk of developing severe sepsis.

**Conclusions:**

AKI is an independent risk factor for severe sepsis. Even patients who recovered from AKI had a high risk of long-term severe sepsis.

## Introduction

Sepsis can cause multiorgan dysfunction, especially of the heart, lung and kidney, causing high morbidity and mortality in hospitalized patients [1]. *Severe sepsis*, defined as sepsis with organ dysfunction, hypoperfusion or hypotension, occurs in about 11% to 27% of ICU patients, with a mortality rate of about 18% to 55% [2-4]. The incidence and severity of sepsis has increased in recent years and has contributed substantially to the disease burden over time [5].

AKI is a common complication, especially in critically ill patients, occurring in 8% of hospitalized patients and in approximately 50% of ICU patients [6]. Sepsis is the most common cause of AKI. Severe sepsis, including septic shock, accounts for almost half of all cases of AKI in the ICU [7,8]. In humans, extrarenal organ dysfunction frequently coexists with AKI, potentiating the already high rates of AKI-associated morbidity and mortality. Unique systemic inflammatory patterns have also been observed for different mechanisms of AKI [9], as the deleterious interaction arises, at least in part, from systemic inflammatory changes, activation of proapoptotic pathways, increases in leukocyte trafficking and dysregulated channel expression [10]. These in turn can lead to long-term sepsis. The aim of this study was to evaluate the incidence of severe sepsis after hospitalization in patients with or without AKI requiring dialysis in a large-scale, population-based administration database in Taiwan. We hypothesized that AKI might be an independent risk factor for developing severe sepsis, regardless of recovery of renal function recovery.

## Materials and methods

### Data sources

The National Health Insurance (NHI) is a nationwide, compulsory, comprehensive health system in Taiwan. Patients were drawn from the NHI Research Database (NHIRD), which was released for research purposes by the National Health Research Institutes, Taipei, Taiwan [11]. The NHIRD, one of the largest databases in the world, covers nearly all (99%) inpatient and outpatient claims for its population of more than 22 million people, and it has been used extensively in previous studies [5,12]. The NHIRD provides encrypted patient identification numbers; age; sex; dates of hospital admission and discharge; medical institutions providing services; *International Classification of Diseases, Ninth Edition, Clinical Modification* (ICD-9-CM), codes of diagnoses and procedures; and outcome at hospital discharge (recovered, died or transferred out).

### Patient selection and definition

**Figure 1:**
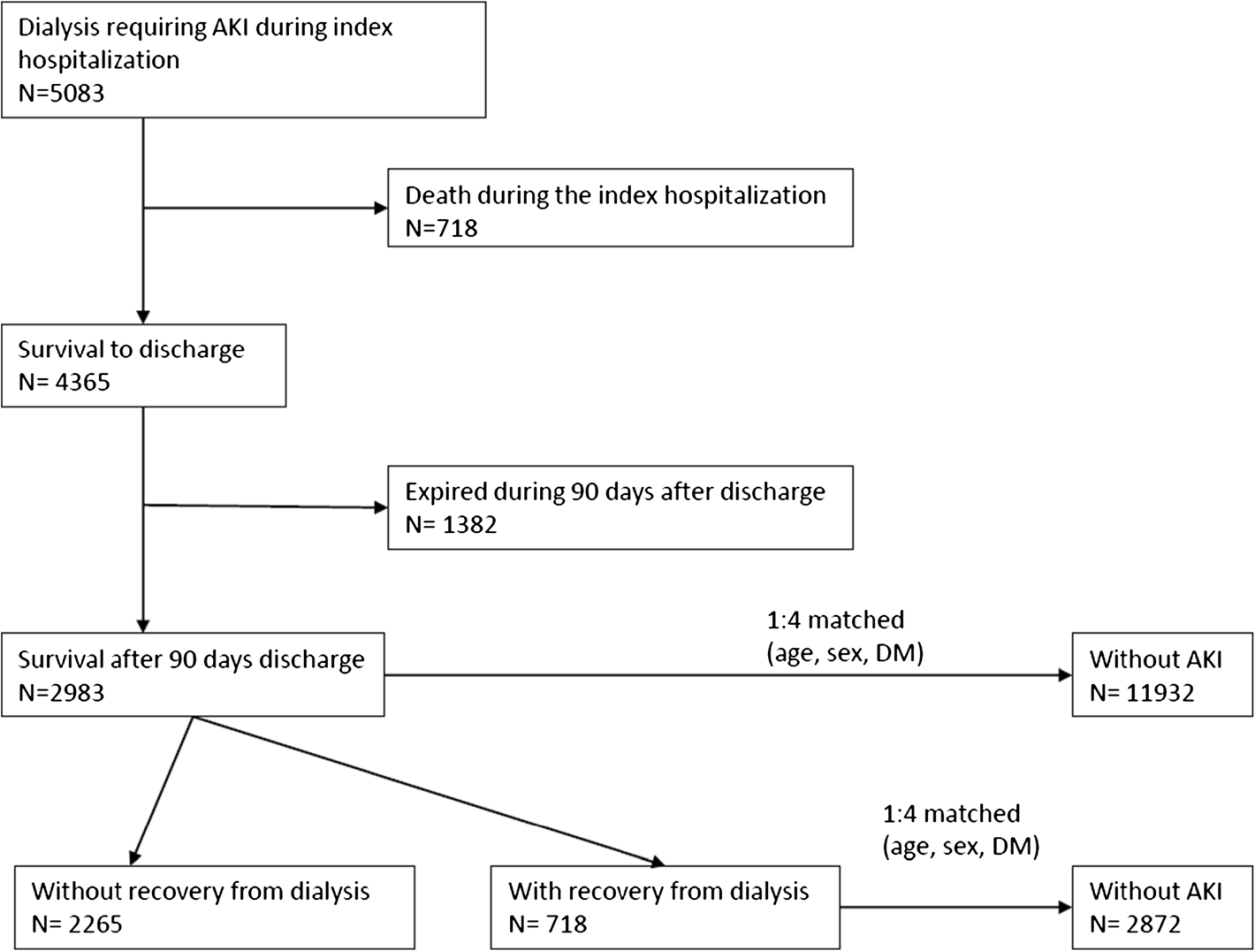
**Flow diaphragm of the study population.** AKI: acute kidney injury.

The NHIRD consists of all the original claim data and registration files of one million individuals from 1999 to 2008. These one million individuals were randomly sampled from the 2000 Registry for Beneficiaries of the Taiwan NHI program and screening comorbidities by using the medical records for one year preceding admission. We linked to diagnostic codes through the hospitalization claims data to identify all episodes of AKI and the corresponding order codes used for the study subjects. We defined the first AKI hospitalization requiring dialysis as the index hospitalization for each individual. Participants who received dialysis during the index hospitalization were included. We excluded individuals who had a previous diagnosis of AKI, had received a kidney transplant or any form of dialysis preceding the index hospitalization AKI (*n* = 6,420), were hospitalized for more than 180 days with AKI (*n* = 36), died during the index hospitalization AKI (*n* = 718) or withdrew from the NHI within three months after discharge (*n* = 1,382). Each AKI patient was then matched with four hospitalized patients without AKI according to age, sex and history of diabetes (Figure 1). Informed consent was originally obtained by the NHRI, and, since patients were anonymous in the present study, informed consent was not required. Also, since the identification numbers of all individuals in the NHRID were encrypted to protect the privacy of the individuals, this study was exempt from a full ethical review by the institutional ethics review board of National Taiwan University Hospital (reference no. 201212021RINC).

### Ascertainment of study outcome

Patients were tracked for outcomes beginning 90 days after discharge and ending at 31 December 2009. The main study outcome was severe sepsis. The method of identification of patients with severe sepsis was similar to that used by Angus *et al*. [3], who selected all acute care hospitalizations with ICD-9-CM codes for both a bacterial or fungal infection process and a diagnosis of acute organ dysfunction. We used codes for acute organ dysfunction as modified by Shen *et al*. [5] (listed in Table 1). *Sepsis* was defined according to the American College of Chest Physicians/Society of Critical Care Medicine (ACCP/SCCM) as systemic inflammatory syndrome in response to infection, which, when associated with acute organ dysfunction, is said to be severe [2]. For patients with more than one hospital admission for severe sepsis during the study period, the first episode of sepsis was included.

**Table 1:** Acute organ dysfunction code for severe sepsis

**Organ dysfunction**	**Codes**	**Code description**
Cardiovascular	458.0	Hypotension, postural
	458.8	Hypotension, specified type, not elsewhere classified
	458.9	Hypotension, arterial, constitutional
	785.5	Shock
	785.51	Shock, cardiogenic
	785.59	Shock, circulatory or septic
	796.3	Hypotension, transient
Respiratory	518.81	Acute respiratory failure
	518.82	Acute respiratory distress syndrome (ARDS)
	518.85	ARDS after shock or trauma
	786.09	Respiratory insufficiency
	799.1	Respiratory arrest
	96.7 (96.71, 96.72)	Ventilator management
	96.04	Endotracheal intubation (emergency procedure)
	93.9	Continuous positive airway pressure
Renal	580.x	Acute glomerulonephritis
	584.x	Acute renal failure
	586	Renal shutdown, renal failure unspecified
	39.95	Hemodialysis
Hepatic	570	Acute hepatic failure or necrosis
	572.2	Hepatic encephalopathy
	573.3	Hepatitis (septic and not elsewhere classified)
	573.4	Hepatic infarction
Neurologic	293	Transient organic psychosis
	348.1	Anoxic brain injury
	348.3	Encephalopathy, acute
	780.01	Coma
	780.09	Altered consciousness, unspecified
	89.14	Electroencephalography
Hematologic	286.2	Disseminated intravascular coagulation
	286.6	Purpura fulminans
	286.9	Coagulopathy
	287.3-5	Thrombocytopenia, primary, secondary or unspecified
	790.92	Abnormal coagulation profile
Metabolic	276.2	Acidosis, metabolic or lactic

### Statistical analyses

For individuals with and those without AKI and dialysis, continuous variables were compared using an unpaired *t*-test and are expressed as mean values with SD. Categorical variables were compared using a *χ*^2^ test and expressed as a percentage. The person-years were calculated from enrollment to outcome, death or the end of the study period. Incidence rates of severe sepsis were calculated for participants with and those without AKI and dialysis, respectively.

Each acute dialysis patient was matched with four non-AKI individuals according to age, sex and history of diabetes. Crude hazard ratios (HRs) with 95% confidence intervals (CIs) were derived from Cox proportional hazards models, and matched individuals without AKI or dialysis constituted the reference group. A Kaplan–Meier curve was generated from unadjusted hazards models. We further adjusted for age, sex, comorbidities, procedure codes and a propensity score in the multivariate models. Subsequent advanced chronic kidney disease (CKD) after discharge was evaluated for its effect on long-term severe sepsis using a time-dependent Cox analysis. The adjusted HR values for severe sepsis were further stratified according to comorbidities.

We used a propensity score approach to account for baseline differences between the AKI groups. Multiple logistic regressions were used to generate propensity scores, which were the predicted probabilities of AKI requiring dialysis. Covariates that were statistically significant in the unadjusted analysis were fit into the model, including age; ICU admission during the index hospitalization; admission frequency; use of mechanical ventilation; comorbidities before admission, such as diabetes mellitus, tumor with metastasis, CKD, chronic obstructive pulmonary disease and congestive heart failure; and comorbidities during index hospitalization, such as those of renal, metabolic, pulmonary or cardiovascular origin. We incorporated this score into a Cox proportional hazards model as a covariate.

In subgroup analysis, each acute dialysis patient with recovery from dialysis was matched with patients without AKI according to age, sex and history of diabetes. A two-sided *P* value less than 0.05 was considered statistically significant. All data were analyzed using R statistical software (version 2.14.1; Free Software Foundation, Inc, Boston, MA, USA).

## Results

A total of 2,983 individuals with AKI requiring dialysis who survived at least 90 days after hospital discharge were identified, and 11,932 individuals without dialysis or AKI were matched. Baseline characteristics between individuals with or without AKI acute dialysis are listed in Table 2. The acute dialysis group had more macrovascular and microvascular disease than the non-AKI group. Among the 2,983 individuals with AKI requiring dialysis, 2,265 patients remained on dialysis and 718 recovered from AKI requiring dialysis after 90 days postdischarge.

**Table 2:** **Baseline characteristics of study population stratified by acute kidney injury**^**a**^

**Patient characteristics**	**Non-AKI group**	**AKI group**	***P*-value**
	**(*N*** **= 11,932)**	**(*N*** **= 2,983)**	
Mean age (years)	62.03 ± 14.84	62.03 ± 14.84	0.995
Males (%)	6,004 (50.3%)	1,501 (50.3%)	0.999
Admission frequency	0.57 ± 1.61	2.28 ± 2.75	<0.001
Charlson Comorbidity Index score	1.67 ± 1.72	3.66 ± 2.17	<0.001
Comorbidities (before admission)			
Myocardial infarction	122 (1%)	89 (3%)	<0.001
Congestive heart failure	426 (3.6%)	549 (18.4%)	<0.001
Peripheral vascular disease	161 (1.3%)	102 (3.4%)	<0.001
Cerebrovascular disease	1,117 (9.4%)	477 (16%)	<0.001
Dementia	204 (1.7%)	87 (2.9%)	<0.001
COPD	1,854 (15.5%)	497 (16.7%)	0.137
Rheumatologic disease	158 (1.3%)	55 (1.8%)	0.038
Peptic ulcer	2,182 (18.3%)	786 (26.3%)	<0.001
Moderate or severe liver disease	1,288 (10.8%)	285 (9.6%)	0.049
Diabetes mellitus	5,973 (50.1%)	1,494 (50.1%)	0.984
Hemiplegia	147 (1.2%)	68 (2.3%)	<0.001
Chronic kidney disease	845 (7.1%)	2,248 (75.4%)	<0.001
Solid tumor	657 (5.5%)	193 (6.5%)	0.047
Tumor with metastasis	198 (1.7%)	40 (1.3%)	0.252
Comorbidities (during index hospitalization)			
Cardiovascular	89 (0.7%)	84 (2.8%)	<0.001
Respiratory	100 (0.8%)	286 (9.6%)	<0.001
Hepatic	96 (0.8%)	36 (1.2%)	0.048
Neurologic	18 (0.2%)	48 (1.6%)	<0.001
Hematologic	56 (0.5%)	29 (1%)	0.002
Metabolic	5 (0%)	98 (3.3%)	<0.001
Operative categories			
Cardiothoracic	78 (0.7%)	51 (1.7%)	<0.001
Upper GI	72 (0.6%)	12 (0.4%)	0.219
Lower GI	128 (1.1%)	20 (0.7%)	0.049
Hepatobiliary	197 (1.7%)	13 (0.4%)	<0.001
ICU admission during index hospitalization	898 (7.5%)	948 (31.8%)	<0.001
Mechanical ventilation	376 (3.2%)	527 (17.7%)	<0.001
Mortality	2603 (21.8%)	1,386 (46.5%)	<0.001
Severe sepsis	1,250 (10.5%)	675 (22.6%)	<0.001

^a^AKI: acute kidney injury; COPD: chronic obstructive pulmonary disease; GI: gastrointestinal.

The mean age of the enrolled participants was 62 ± 14.8 years, and 50.3% were men. Compared with non-AKI participants, those with acute dialysis had a higher mean Charlson Comorbidity Index score (3.7 ± 2.2 vs. 1.7 ± 1.7) and more comorbidities before the index hospitalization, including cardiovascular, peripheral vascular and cerebrovascular disease. Participants with acute dialysis had a much higher prevalence of underlying CKD (75.4% vs. 7.1%) than did non-AKI patients. During their hospitalization, patients with acute dialysis were more likely to receive mechanical ventilation (17.7 vs. 3.2%; *P* < 0.001), had higher long-term mortality (46.5 vs. 21.8%; *P* < 0.001) and developed severe sepsis more often (22.6 vs. 10.5%) than did patients without AKI. The respective rates for severe sepsis were 22.6% and 10.5%.

**Figure 2:**
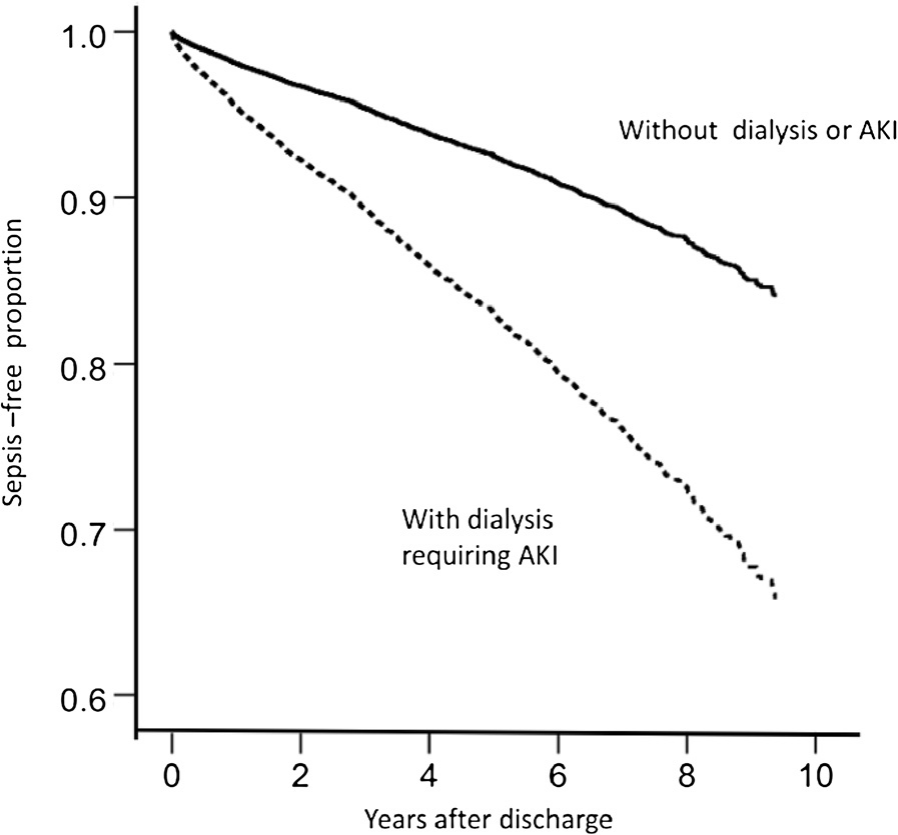
**Kaplan–Meier curve for incidence of severe sepsis after discharge for patients with or without acute kidney injury.** AKI: acute kidney injury.

**Figure 3:**
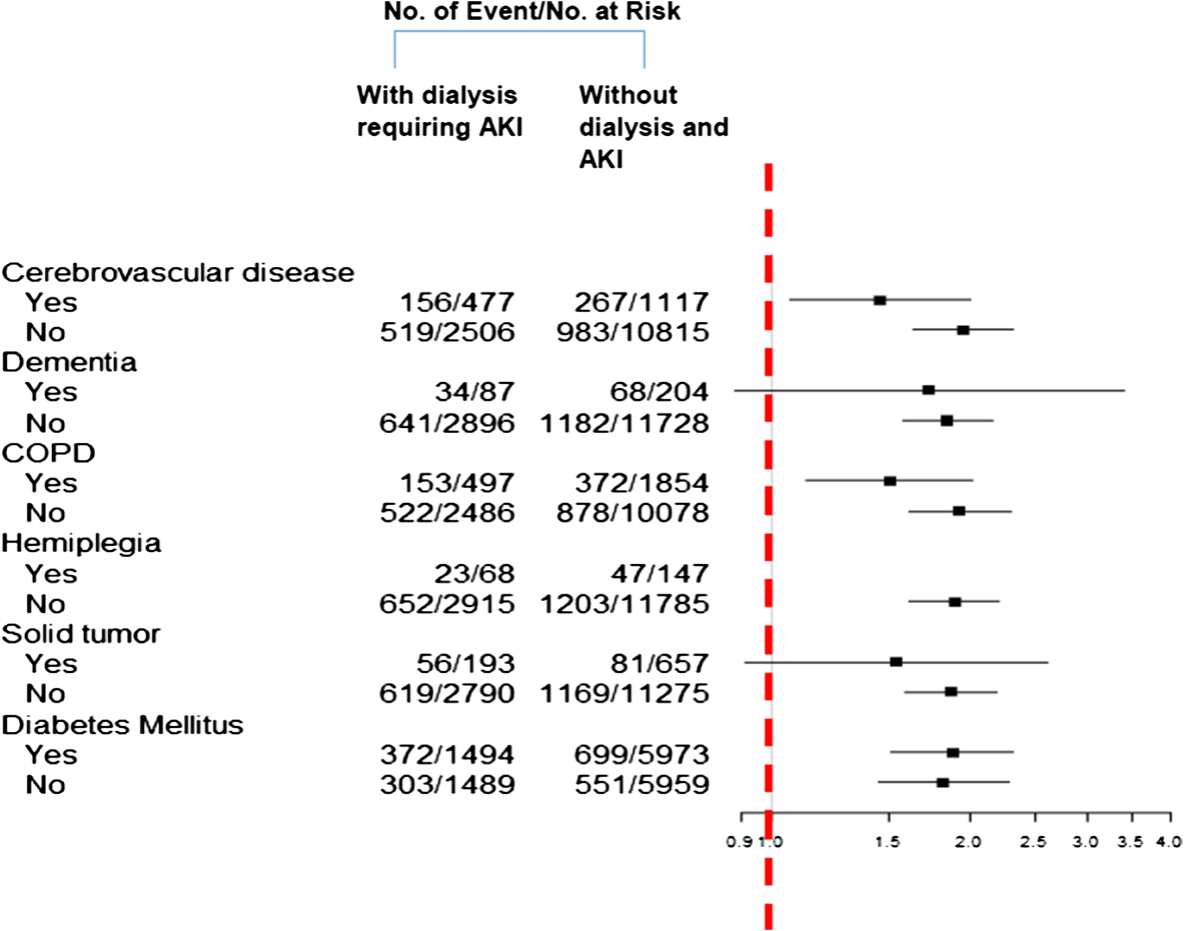
**Risk of severe sepsis associated with acute kidney injury at index hospitalization by participant characteristics.** AKI: acute kidney injury.

After a median follow-up period of 3.96 years, the incidence rates of severe sepsis were 6.84 and 2.32 per 100 person-years among individuals with and those without AKI and dialysis in the index hospitalization, respectively, resulting in a HR of 2.87 (95% CI, 2.62 to 3.16; *P* < 0.001). The propensity score–adjusted HR was 1.83 (95% CI, 1.57 to 2.13; *P* < 0.001). After multivariable adjustment, patients with AKI requiring dialysis were at a higher risk than patients in the non-AKI group for developing *de novo* severe sepsis (HR: 1.99 (95% CI: 1.71 to 2.31); *P* < 0.001) (Table 3). This model had good validity with a C-index concordance statistic of 0.79 and an adjusted generalized *R*^2^ of 0.12. The Cox proportional hazards model is plotted in Figure 2. In time-dependent analysis, the AKI group requiring dialysis had a higher risk of developing severe sepsis than the non-AKI group in the first year and decreased gradually with time (one-year HR: 3.44 (95% CI: 2.59 to 4.56), two-year HR: 2.26 (95% CI: 1.83 to 2.78] and three-year HR: 2.05 (95% CI: 1.70 to 2.48), respectively) (Table 4). For participants with and those without recovery from AKI and dialysis, the tendency for the development of severe sepsis did a 95% CI of 0.9 to 1.34 (*P* = 0.457). The increased risk of developing long-term severe sepsis following hospitalization complicated by AKI requiring dialysis was consistent across patient subgroups, including cerebrovascular disease, chronic obstructive pulmonary disease, hemiplegia and diabetes mellitus (Figure 3).

**Table 3:** **Crude and adjusted hazard ratios of severe sepsis for acute kidney injury status**^**a**^

**Analysis model**	**AKI vs. non-AKI**		**Recovery vs. non-AKI**	
	**HR (95% CI)**	***P*-value**	**HR (95% CI)**	***P*-value**
Univariate	2.87 (2.62 to 3.16)	<0.001	2.80 (2.34 to 3.34)	<0.001
Multivariate				
Model 1	1.83 (1.57-2.13)	<0.001	1.48 (1.06 to 2.06)	0.02
Model 2	1.99 (1.71 to 2.31)	<0.001		
Model 3			1.58 (1.15 to 2.16)	<0.001
Model 4	1.95 (1.67 to 2.28)	<0.001		

^a^AKI: acute kidney injury; HR: hazard ratio. Model 1: Adjusted for age, sex and propensity score.

Model 2: Adjusted for age, sex, propensity score, ICU admission, admission frequency, Charlson Comorbidity Index score, comorbidities before index hospitalization (cerebrovascular disease, dementia, chronic obstructive pulmonary disease, hemiplegia and metastatic tumor) and respiratory and hematologic comorbidities during index hospitalization. Model 3: Adjusted for age, sex, propensity score, admission frequency, Charlson Comorbidity Index score, comorbidities before index hospitalization (peptic ulcer and diabetes mellitus) and cardiovascular and respiratory comorbidities during index hospitalization. Model 4: Model 2 adjusted with subsequent advanced CKD after hospital discharge as a time-varying covariate. *Advanced CKD* is defined as patients with serum creatinine levels above 6 mg/dl and a concomitant erythropoiesis-stimulating agent prescription.

**Table 4:** **Time-dependent analysis of the risk of developing severe sepsis**^**a**^

**Model**	**AKI vs. non-AKI**		**Recovery vs. non-AKI**	
	**HR (95% CI)**	***P*-value**	**HR (95% CI)**	***P*-value**
Model 5	3.44 (2.59 to 4.56)	<0.001	1.61 (1.00 to 2.59)	0.049
(one year of follow-up)				
Model 6	2.26 (1.83 to 2.78)	<0.001	1.61 (1.09 to 2.37)	0.017
(two years of follow-up)				
Model 7	2.05 (1.70 to 2.48)	<0.001	1.68 (1.18 to 2.40)	0.004
(three years of follow-up)				

^a^AKI: acute kidney injury. (a) AKI group vs. non-AKI group. (b) Recovery group vs. non-AKI group). Model 5a: Adjusted for age, propensity score, admission frequency, comorbidities before index hospitalization (chronic kidney disease (CKD), cerebrovascular disease, dementia, chronic pulmonary disease and metastatic tumor), hematologic comorbidities during index hospitalization and advanced CKD. *Advanced CKD* is defined as patients with serum creatinine levels above 6 mg/dl and a concomitant erythropoiesis-stimulating agent prescription. Model 5b: Adjusted for age, propensity score, admission frequency, Charlson Comorbidity Index score, comorbidities before index hospitalization (cerebrovascular disease and peptic ulcer) and cardiovascular and respiratory comorbidities during index hospitalization. Model 6a: Adjusted for age, propensity score, Charlson Comorbidity Index score, admission frequency, comorbidities before index hospitalization (CKD, cerebrovascular disease, dementia and chronic obstructive pulmonary disease), metabolic and respiratory comorbidities during index hospitalization and advanced CKD. Model 6b: Adjusted for age, propensity score, admission frequency, Charlson Comorbidity Index score, comorbidities before index hospitalization (cerebrovascular disease and peptic ulcer) and cardiovascular, respiratory and hepatic comorbidities during index hospitalization. Model 7a: Adjusted for age, propensity score, Charlson Comorbidity Index score, admission frequency, comorbidities before index hospitalization (CKD, cerebrovascular disease, dementia and chronic obstructive pulmonary disease) and metabolic, hematologic and respiratory comorbidities during index hospitalization. Model 7b: Adjusted for age, propensity score, admission frequency, Charlson Comorbidity Index score, comorbidities before index hospitalization (cerebrovascular disease and peptic ulcer) and respiratory comorbidities during index hospitalization.

In a subgroup analysis, we compared individuals who recovered from AKI requiring dialysis with non-AKI individuals. The crude HR and propensity score–adjusted HRs were 2.80 (95% CI: 2.34 to 3.34; *P* < 0.001) and 1.48 (95% CI: 1.06 to 2.06; *P* = 0.02), respectively. The recovery group was at a higher risk for severe sepsis than the non-AKI group after multivariable adjustment for the covariates listed in Table 3 (HR: 1.58 (95% CI: 1.15 to 2.16); *P* < 0.001). This model also showed good validity, with a C-index concordance statistic of 0.78 and an adjusted generalized *R*^2^ of 0.15.

## Discussion

AKI is an independent predictor of increasing mortality in critically ill patients [13,14]. Our study demonstrates that AKI requiring dialysis is associated with subsequent long-term severe sepsis. After a median follow-up of 3.96 years, the incidence of severe sepsis in the AKI group was about threefold the incidence of severe sepsis in the non-AKI group. In a subgroup analysis, we found that, even in individuals who recovered from AKI and dialysis, the incidence of severe sepsis was 58% higher than that in individuals without AKI, suggesting that AKI *per se* is an independent risk factor for developing severe sepsis, regardless of recovery from AKI. The prevalence rate of severe sepsis was 2.32 per 100 person-years in the non-AKI group, which is consistent with the national estimate of 2.26 per 100 person-years in hospital patients in the United States [3].

The prevalence of sepsis after AKI has been underestimated, and, until now, only a few studies have emphasized the effect of AKI on the incidence of sepsis. For example, a cohort study of contrast-induced AKI illustrated that 45% of patients developed sepsis subsequently during hospitalization, but the long-term development of sepsis was not reported [15]. Another study retrospectively investigated the incidence of infection in critically ill patients with AKI and found that 80% of critical ill patients with AKI developed infections during hospitalization [16]. Of all episodes of infection, 46.2% occurred before, 40.3% during and 13.4% after discontinuation of renal replacement therapy. Single-center enrollment or a small sample size limits the robustness of studies, however, and the definition of sepsis is often overestimated. Recently, in a multicenter, observational study, Mehta *et al*. analyzed data from 618 critically ill patients in the ICU and examined the relationship between sepsis and AKI [17]. The mortality rate for patients with who developed sepsis after AKI was higher than that for sepsis-free patients (44% vs. 21%; *P* < 0.001) and similar to that for patients with sepsis preceding AKI (48% vs. 44%; *P* = 0.41). By using comprehensive administrative data in Taiwan in our present study, we have provided strong evidence that AKI is an independent risk factor for developing severe sepsis, even when it occurs a long time after index hospitalization. Furthermore, although dialysis was an important predictor of sepsis after AKI diagnosis, our results show that patients who recovered from dialysis still had a higher risk of subsequent severe sepsis, suggesting that chronic dialysis itself is not the crucial source of post-AKI sepsis in these patients.

Patients in the ICU are dying as a result of AKI, but not simply from AKI. Experimental and small observational studies have shown that AKI negatively affects immunity and is associated with higher rates of infection [18]. AKI patients frequently develop a vicious cycle of immune dysfunction, sepsis and multiorgan failure. Indeed, severe sepsis is currently the major cause of AKI in the United States [19]. The host response to sepsis involves an inflammatory response which activates innate immunity. If this persists, the immune response will lead to a release of a multitude of proinflammatory products, which frequently cause organ dysfunction, including kidney failure [20-26]. Our finding that patients with AKI were fragile and were more likely to develop severe sepsis reinforces the fact that acute renal dysfunction plays a crucial immunomodulatory role in individuals in stressed states. Also, AKI has been proved to facilitate organ cross-talk in the heart, brain, lungs, liver and other organs via the relevant biological pathways [27,28]. Multiple end-stage organ injury after AKI may worsen the severity of infection in severe sepsis.

Though renal function has been thought to be reversible after AKI, recent studies have provided evidence that AKI increases the risk of CKD and end-stage renal disease requiring dialysis [7,29,30]. A systematic review and meta-analysis showed a higher risk of CKD and chronic dialysis in patients after AKI [31]. Clinical studies have also demonstrated that CKD attenuated immunity and subsequently increased the risk of pneumonia, influenza and associated sepsis [32]. Thus, increased risk of severe sepsis, even among the recovery group, with subsequent CKD and impaired immunity is reasonable. In our analysis, we further adjusted for advanced CKD status represented by erythropoietin use, and we found that the AKI group requiring dialysis was still at a higher risk for developing severe sepsis (HR: 1.95 (95% CI: 1.67 to 2.28); *P* < 0.001), suggesting that patients with AKI are still at risk of developing severe sepsis after removal of the uremia effect.

Our study has several limitations. First, when using administrative databases, the diagnosis of AKI, severe sepsis and other comorbidities are totally based on ICD and procedure codes, so misclassification inevitably occurs. However, the misclassification is often nondifferential and the outcome difference is toward null. In a recent validation study, a diagnostic code for AKI plus a procedure code for dialysis had a sensitivity and specificity of more than 90% for capturing patients with AKI requiring dialysis [33]. Furthermore, the NHIB in Taiwan has randomly reviewed medical charts and audited all medical charges [34]; hence, the data quality is trustworthy. Second, individual information such as smoking, drinking and site of infection was not available through administrative data in the NHIRD. We were also unable to obtain detailed laboratory data, such as serum creatinine level or urinary protein excretion. There may be residual confounding factors caused by these unknown data. However, these factors have less effect on our outcome than the factors we adjusted for, and the robustness of the association between AKI and long-term severe sepsis is largely unaffected.

## Conclusions

In this study, we have demonstrated that AKI is an independent risk factor for the development of long-term severe sepsis. The incidence of severe sepsis is threefold in patients with AKI requiring dialysis compared with non-AKI patients. Our study further indicates that individuals who recovered from AKI and dialysis still had a higher risk than non-AKI patients of developing severe sepsis. Therefore, all AKI patients requiring dialysis, regardless they recover, should be considered at risk for severe sepsis and should be aggressively managed for infection prevention. Because of the high mortality in severe sepsis, awareness of the high risk of severe sepsis after AKI should be raised in outpatient clinical practice.

## Key messages

• Sepsis has been a factor in patients with AKI; however, the long-term risk of severe sepsis in patients after AKI requiring dialysis may be overlooked.

• We have revealed that AKI is an independent risk factor for the development of long-term severe sepsis. The incidence of severe sepsis in the AKI group was about threefold the incidence of severe sepsis in the non-AKI group after a median of four years follow-up.

• We also revealed that even patients who recovered from AKI had a high risk of long-term severe sepsis. Therefore, we should view AKI as not only a self-limiting acute disease but also a long-lasting immunomodulatory disorder.

• We need to raise the awareness of the high risk of severe sepsis after AKI in outpatient clinical practice. All AKI patients requiring dialysis, regardless of whether they recover, should be aggressively managed for infection prevention.

## Abbreviations

ACCP/SCCM: American College of Chest Physicians/Society of Critical Care Medicine; AKI: Acute kidney injury; CI: Confidence interval; CKD: Chronic kidney disease; COPD: Chronic obstructive pulmonary disease; DM: Diabetes mellitus; HR: Hazard ratio; ICD-9-CM: *International Classification of Diseases, Ninth Edition, Clinical Modification*; NHI: National Health Insurance; NHIRD: National Health Insurance Research Database.

## Competing interests

The authors declare that they have no competing interests.

## Authors’ contributions

TSL conceived the study, participated in data collection, performed statistical analysis, interpreted the results and wrote the manuscript. CYW, SCP, TMH, CFL and VCW participated in data collection and manuscript revision. MCL and CHW participated in data collection. VCW and KLC conceived the study and participated in manuscript revision. All authors read and approved the final manuscript.

## Authors’ information

The National Taiwan University Hospital Study Group for Acute Renal Failure (NSARF) includes Wen-Je Ko, Vin-Cent Wu, Chun-Fu Lai, Tao-Min Huang, Tai-Shuan Lai, Yung-Ming Chen, Chih-Chung Shiao, Wei-Shun Yang, Wei-Jie Wang, Likwang Chen, Cheng-Yi Wang, Pei-Chen Wu, Pi-Ru Tsai, Yu-Chang Yeh, Fu-Chang Hu and Kwan-Dun Wu.
